# Supplementary material for: Linewidth-related bias in modelled concentration estimates from GABA-edited 1H-MRS
Source: bioRxiv. 2024 Feb 28:2024.02.27.582249. Preprint. [Version 1] doi: 10.1101/2024.02.27.582249 (PMC10925149; doi:10.1101/2024.02.27.582249)
Supplement: Supplement 1 [file media-1.pdf]

Supplementary Material

A MRSinMRS checklist 2
B Acquisition Details 3
C Supplementary Details: In-vivo data 4
D Supplementary Details: Synthetic Data 10
D.1 Synthetic data generation 10
D.2 Outcomes from fits to synthetic data 11
E Exploratory Analysis #1: spant baseline modelling 13
E.1 Background and Methods 13
E.2 Results and Discussion 14
F Exploratory Analysis #2: FSL-MRS baseline modelling, optimization and lineshape 17
F.1 Background and Methods 17
F.2 Results and Discussion 18
G Exploratory Analysis #3: LCModel baseline knot spacing 22
G.1 Background and Methods 22
G.2 Results and Discussion 22
H Supplementary Methods 24
H.1 Difference spectrum modelling 24
H.1.1 FSL-MRS 24
H.1.2 Gannet 25
H.1.3 LCModel 25
H.1.4 Osprey 26
H.1.5 spant 26
H.1.6 Tarquin 27
H.2 Creatine Reference: Edit-OFF sub-spectrum modelling 28
H.2.1 FSL-MRS 28
H.2.2 Gannet 29
H.2.3 LCModel 29
H.2.4 Osprey 29
H.2.5 spant 29
H.2.6 Tarquin 30

A MRSinMRS checklist

Site (name or number)	[GPS](1-10)
**1. Hardware**	
a. Field strength [T]	3 T
b. Manufacturer	See sup. table 2
c. Model (software version if available)	
d. RF coils: nuclei (transmit/receive), number of channels, type, body part	
e. Additional hardware	N/A
**2. Acquisition**	
a. Pulse sequence	MEGA-PRESS (GABA+ editing; see sup. table 2 for variants)
b. Volume of interest (VOI) locations	Medial parietal lobe
c. Nominal VOI size [cm3,mm3]	30 x 30 x 30 mm3
d. Repetition time (T_R), echo time (T_E) [ms, s]	T_R = 2000 ms, T_E = 68 ms
e. Total number of excitations or acquisitions per spectrum<br>In time series for kinetic studies<br>i. Number of averaged spectra (NA) per time point<br>ii. Averaging method (eg block-wise or moving average)<br>iii. Total number of spectra (acquired/in time series)	Up to 320 averages (160 ON and 160 OFF transients)
f. Additional sequence parameters (spectral width in Hz, number of spectral points, frequency offsets)<br>If STEAM:, mixing time (T_M)<br>If MRSI: 2D or 3D, FOV in all directions, matrix size, acceleration factors, sampling method	See sup. table 2 for spectral width and number of points.<br>15 ms editing pulses at 1.9 ppm (ON) and 7.46 ppm (OFF)
g. Water suppression method	See sub. table 2
h. Shimming method, reference peak, and thresholds for “acceptance of shim” chosen	See sup. table 2; acceptance criteria varied across sites
i. Triggering or motion correction method (respiratory, peripheral, cardiac triggering, incl. device used and delays)	N/A
**3. Data analysis methods and outputs**	
a. Analysis software	FSL-MRS 1.1.1<br>Gannet 3.1<br>Osprey 1.0.1.1<br>LCModel 6.3-1P<br>spant 1.13.9<br>Tarquin 4.3.11 (local patch)
b. Processing steps deviating from quoted reference or product	Filtering to experimentally adjust SNR and linewidth, see section 2.1.1
c. Output measure (eg absolute concentration, institutional units, ratio), processing steps deviating from quoted reference or product	Water-referenced estimates (with adjustment for tissue content); see section 2.4
d. Quantification references and assumptions, fitting model assumptions	Standardised basis set where applicable, incorporating 3.0 ppm macromolecule component; see sections 2.2, 2.4
**4. Data quality**	
a. Reported variables (SNR, linewidth (with reference peaks))	SNR and Linewidth from NAA_{diff}
b. Data exclusion criteria	R1: Correlation with normalized mean spectrum < 0.5 across metabolite range, or deviating from group mean by > 3 SD<br>R2: SNR < 80 (NAA_{diff}) or FWHM linewidth > 10 Hz (NAA_{diff})<br>See section 2.1.2
c. Quality measures of postprocessing model fitting (eg CRLB, goodness of fit, SD of residual)	R3: Strong outliers (> 5 x median absolute deviation), see 2.1.2<br>%SD across variants based on same dataset;<br>% mean signed and abs. errors (%MSE, %MAE) for synthetic data
d. Sample spectrum	See Sup Figure 1

Supplementary Table 1: MRSinMRS checklist[1] summarising key details of the MRS acquisition

B Acquisition Details

Site	N	Age (years±SD)	Sex F / M	Scanner vendor and model	Software release	Tx/Rx hardware	MEGA-PRESS sequence variant	Phase cycling	Editing inter-leaving	B0 shimming approach	Water suppr.	Spectral width (Hz)	Data points
MRSinMRS				1b,c	1c	1d	2a			2h	2g	2f	2f
G1	7	22.9±3.7	4 / 3	GE Discovery MR750w	DV25	Body coil/32-ch head coil	Interleaved sequence	2	2	Double-echo GRE	CHESS	5000	4096
G4	12	25.6±4.5	6 / 6	GE Discovery MR750	DV25	Body coil/8-ch head coil	ATSM patch	8	1	Double-echo GRE	CHESS	5000	4096
G5	12	25.5±3.7	5 / 7	GE Discovery MR750	DV25	Body coil/32-ch head coil	ATSM patch	8	1	Double-echo GRE	CHESS	2000	2048
G6	12	24.3±4.2	6 / 6	GE Signa HDx	HD16	Body coil/8-ch head coil	ATSM patch	2	2	Double-echo GRE	CHESS	2000	2048
G7	12	28.1±4.0	6 / 6	GE Discovery MR750	DV24	Body coil/8-ch head coil	ATSM patch	8	1	Double-echo GRE	CHESS	2000	2048
G8	12	29.7±2.1	6 / 6	GE Discovery MR750	DV24	Body coil/8-ch head coil	ATSM patch	8	1	Double-echo GRE	CHESS	2000	2048
**All G**	**67**	**26.2±4.3**	**33 / 34**										
P1	9	25.0±3.7	4 / 5	Philips Achieva	R5.1.7	Body coil/32-ch head coil	JHU patch	16	1	PB-auto	VAPOR	2000	2048
P3	12	25.1±2.9	6 / 6	Philips Achieva	R3.2.2	Body coil/32-ch head coil	JHU patch	16	1	PB-auto	VAPOR	2000	2048
P4	12	29.2±3.1	5 / 7	Philips Ingenia CX	R5.1.7	Body coil/32-ch head coil	JHU patch	16	1	PB-auto	MOIST	2000	2048
P5	12	24.9±4.3	7 / 5	Philips Achieva TX	R5.1.7	Body coil/32-ch head coil	JHU patch	16	1	PB-auto	MOIST	2000	2048
P6	8	23.1±2.4	3 / 5	Philips Achieva	R3.2.3	Body coil/8-ch head coil	JHU patch	16	1	PB-auto	MOIST	2000	2048
P7	12	27.3±3.7	7 / 5	Philips Ingenia	R5.1.8	Body coil/32-ch head coil	JHU patch	16	1	PB-auto	VAPOR	2000	2048
P8	12	23.6±3.7	6 / 6	Philips Ingenia CX	R5.1.8	Body coil/32-ch head coil	JHU patch	16	1	PB-auto	MOIST	2000	2048
P9	12	23.2±2.0	5 / 7	Philips Achieva	R5.1.7	Body coil/32-ch head coil	JHU patch	16	1	PB-auto	VAPOR	2000	2048
P10	12	25.8±4.6	6 / 6	Philips Ingenia	R5.1.9	Body coil/15-ch head coil	JHU patch	16		PB-auto	MOIST	2000	2048
**All P**	**101**	**25.4±3.9**	**49 / 52**										
S1	12	25.7±3.7	6 / 6	Siemens Trio	VB17	Body coil/32-ch head coil	WIP (529)	16	1	3D-DESS + manual	CHESS	4000	4096
S3	12	31.6±3.4	9 / 3	Siemens Prisma	VD13	Body coil/20-ch head/neck coil	WIP (859D)	16	1	FAST(EST) MAP	WET	4000	4096
S5	12	26.5±3.7	6 / 6	Siemens Trio	VB17	Body coil/12-ch head coil	WIP (529)	16	1	3D-DESS	CHESS	4000	4096
S6	6	26.2±2.0	1 / 5	Siemens Trio	VB17	Body coil/32-ch head coil	WIP (529)	16	1	FAST(EST) MAP	WET	4000	4096
S8	12	24.0±3.5	11 / 1	Siemens Prisma	VE11	Body coil/64-ch head coil	WIP (859G)	16		3D-DESS	WET	4000	4096
**All S**	**54**	**26.9±4.3**	**33 / 21**										
**Total**	**222**	**26.0±4.1**	**115 / 107**										

Supplementary Table 2: Basic demographics, hardware and software parameters for the constituent datasets; reproduced from previously published work [2].

C Supplementary Details: In-vivo data

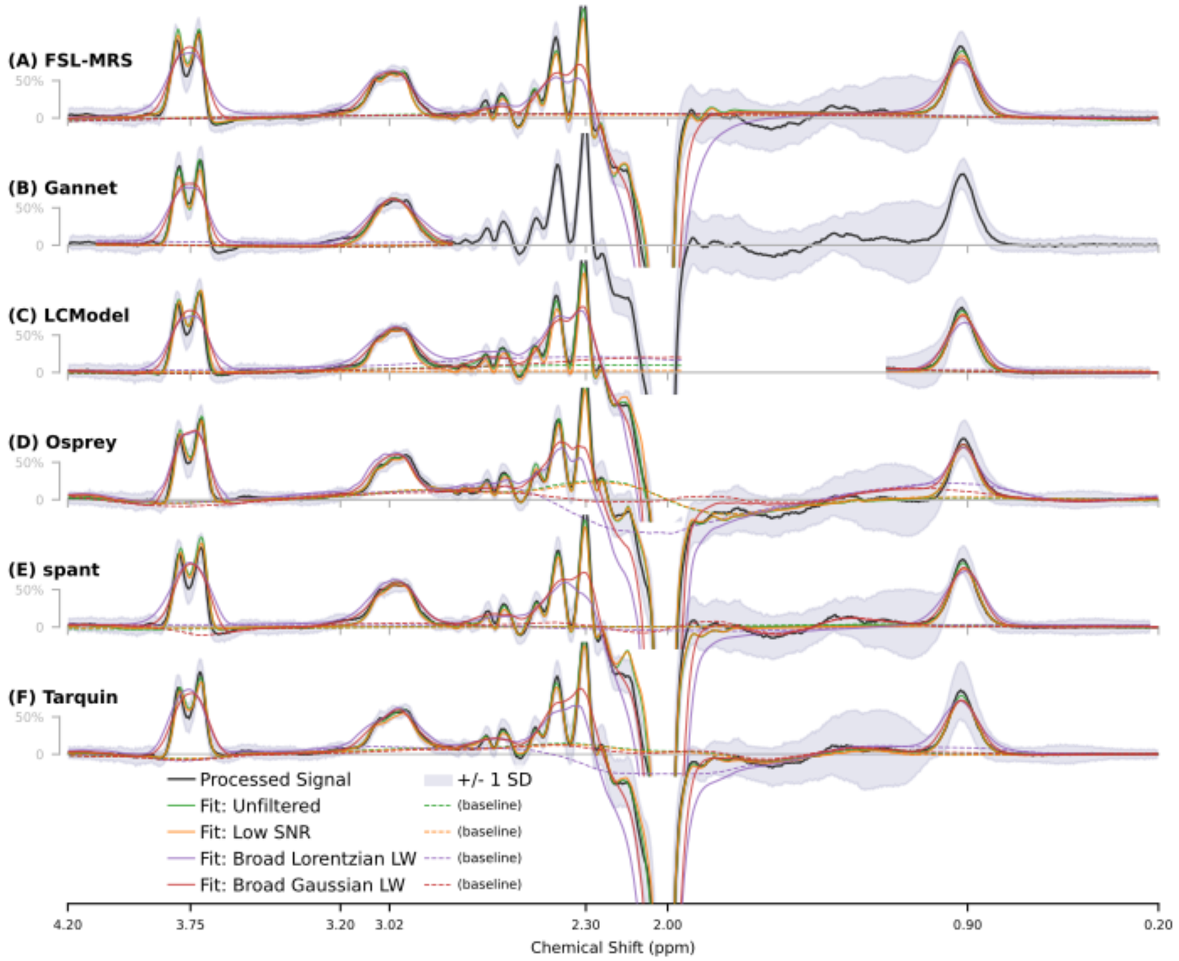

Supplementary Figure 1 Group-average fit by each algorithm, to original (unfiltered) data, low SNR and high linewidth variants

Site	SNR	Linewidth (Hz)	Site	SNR	Linewidth (Hz)
G1	142 [124; 206]	5.64 [5.11; 7.33]	P1	222 [150; 306]	4.71 [4.01; 5.59]
G4	133 [104; 156]	6.74 [5.22; 7.86]	P10	104 [85.9; 129]	5.65 [4.32; 6.92]
G5	167 [118; 193]	5.91 [4.64; 7.27]	P3	142 [109; 176]	5.00 [4.21; 6.3]
G6	141 [127; 188]	5.46 [4.72; 6.53]	P4	242 [202; 273]	4.50 [4.11; 4.9]
G7	133 [97.2; 159]	6.19 [5.51; 8.19]	P5	196 [168; 271]	5.00 [4.27; 5.55]
G8	120 [82.9; 150]	5.59 [4.73; 6.61]	P6	151 [102; 193]	4.62 [4.39; 6.01]
S1	221 [165; 305]	6.17 [4.32; 8.77]	P7	175 [155; 199]	7.61 [5.81; 8.87]
S3	160 [84.3; 232]	4.97 [4.17; 5.81]	P8	257 [189; 308]	4.71 [3.69; 5.97]
S5	166 [121; 193]	5.52 [4.76; 9.41]	P9	216 [172; 255]	4.69 [4.05; 5.18]
S6	271 [190; 289]	5.59 [4.41; 5.95]			
S8	240 [167; 264]	4.49 [4.2; 4.78]	**All**	**171 [104; 273]**	**5.33 [4.25; 7.61]**

Supplementary Table 3 : Basic quality metrics for incoming data (before filtering), per site – expressed as median [5, 95]-percentiles, derived from tNAA measured on the difference spectrum.

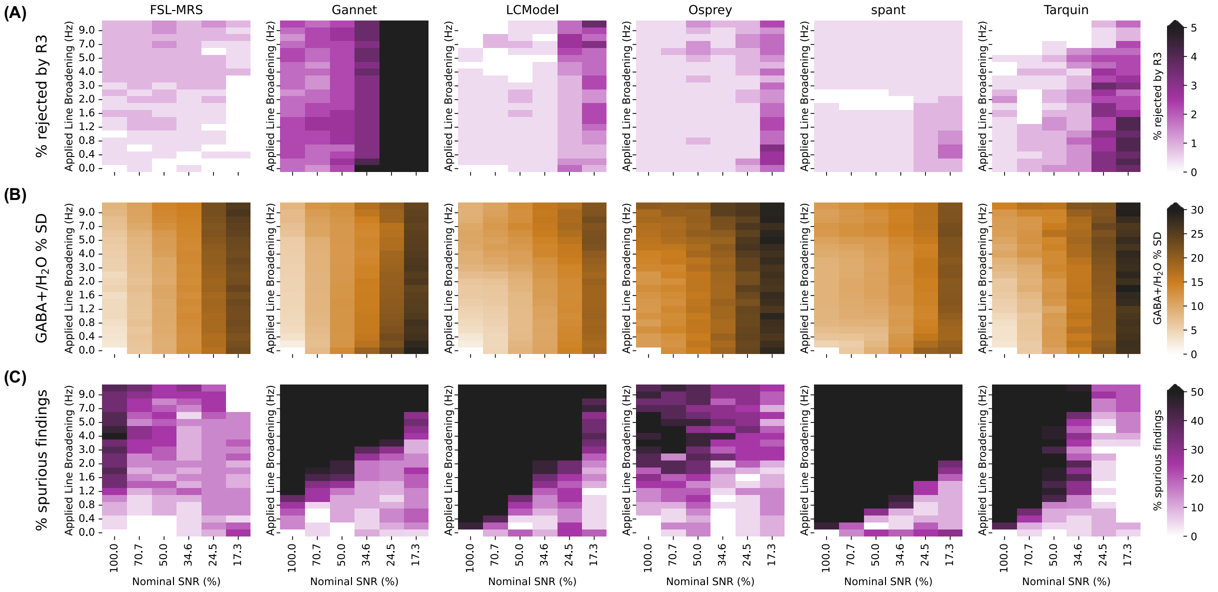

Supplementary Figure 2 : Rejection rate according to R3 (A), standard deviation of GABA+ estimates (B), and rate of "spurious findings": false-positive statistically significant difference in mean outcome, on a per-site basis (C), given the applied change in SNR/LW for ***Lorentzian*** *linebroadening*

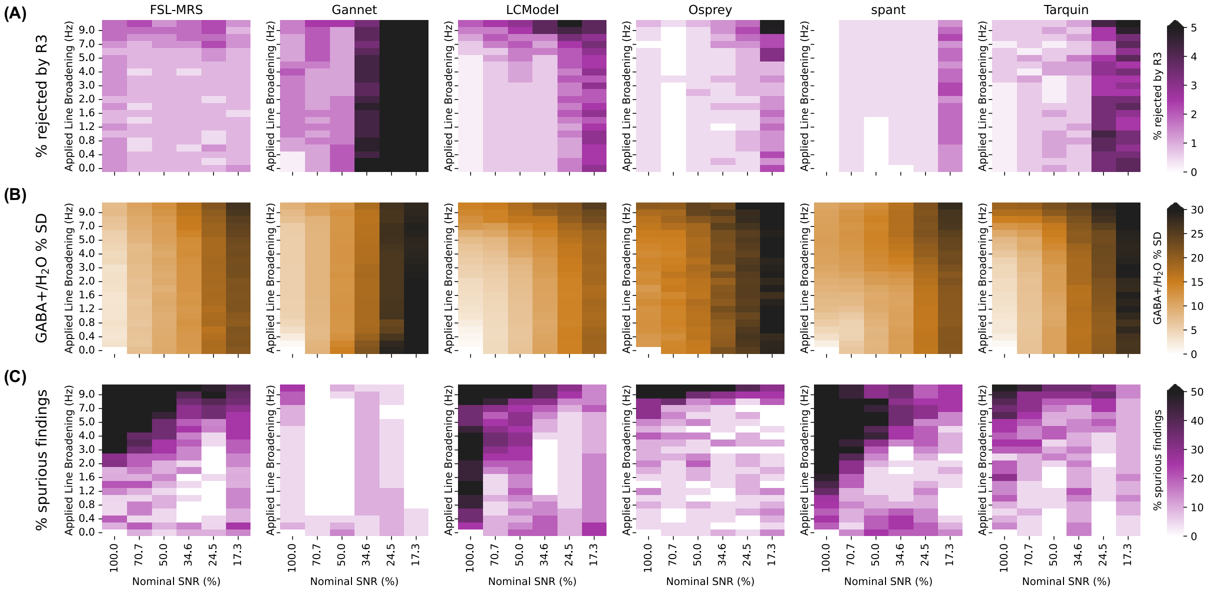

Supplementary Figure 3 Rejection rate according to R3 (A), standard deviation of GABA+ estimates (B), and rate of "spurious findings": false-positive statistically significant difference in mean outcome, on a per-site basis (C), given the applied change in SNR/LW for ***Gaussian*** *linebroadening*

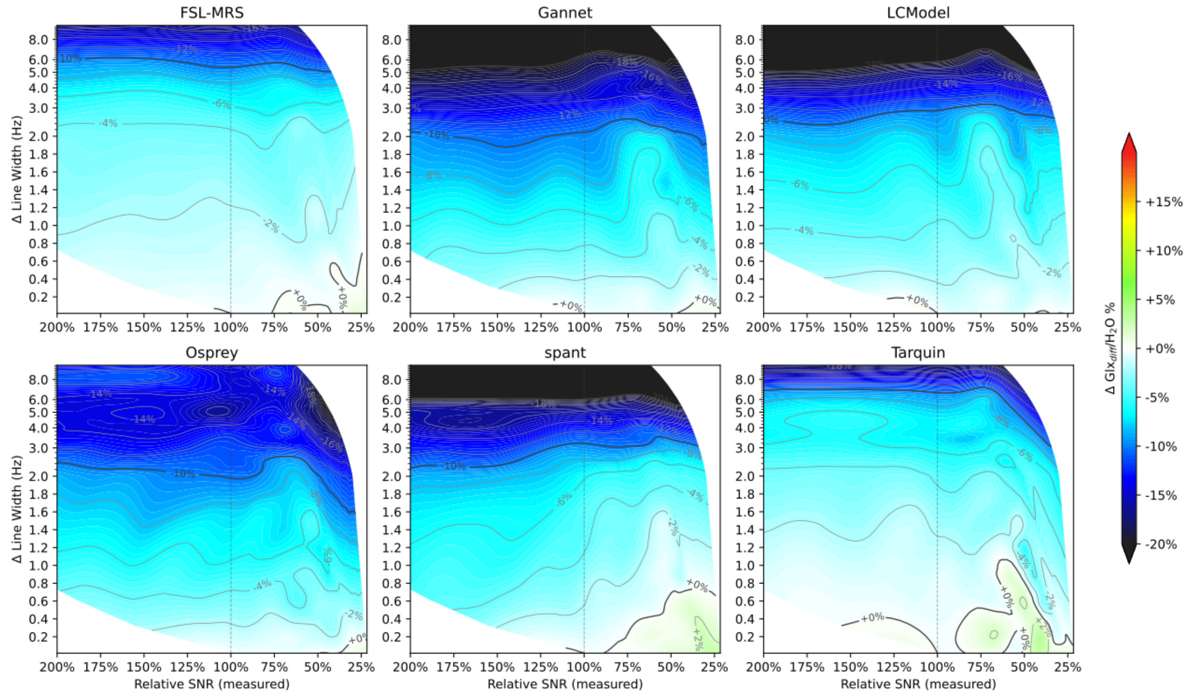

Supplementary Figure 4 Relative Glx estimate as a function of Lorentzian linebroadening and SNR, for each of the algorithms assessed

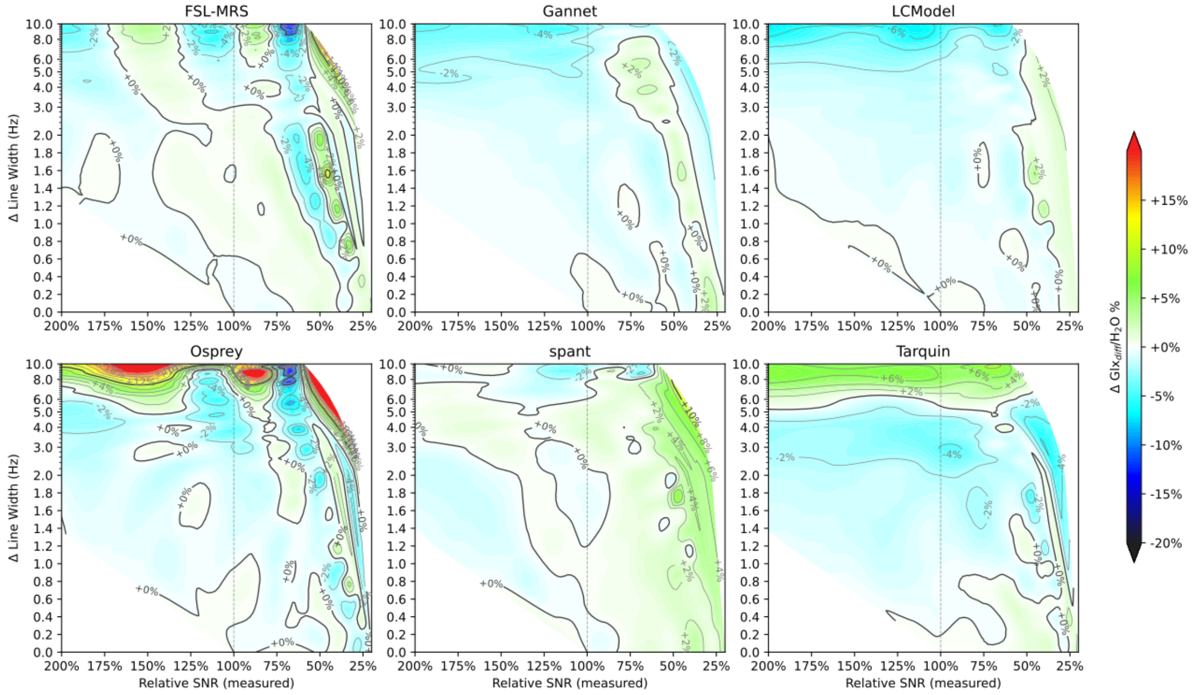

Supplementary Figure 5 Relative Glx estimate as a function of Gaussian linebroadening and SNR, for each of the algorithms assessed

		Lorentzian LB											Gaussian LB										
Line Broadening								0 Hz	3 Hz	10 Hz	all								0 Hz	3 Hz	10 Hz	all	
Relative SNR		100%	71%	50%	35%	24%	17%				all		100%	71%	50%	35%	24%	17%				all	
Algorithm	VPC											Median											Median
FSL-MRS	Subject	87,3	89	91,3	92,5	93,5	90,9	31,5	33,6	38,3	40,4	88,15	84,5	88,3	88,1	90,5	92,3	93,8	34,9	35,1	38,4	45,2	86,3
	LB	2,2	1,6	1,4	1	0,7	1,3				1,1	1,3	6,9	5,5	5	3	1,7	1,2				2,6	3
	SNR							3,2	0,2	0	0,5	0,35							2,2	0,3	0,1	0,6	0,45
	Resid	10,5	9,4	7,3	6,4	5,9	7,9	65,3	66,2	61,7	58	9,95	8,6	6,2	7	6,5	6	5	62,9	64,5	61,4	51,5	7,8
Gannet	Subject	55	61,8	73,5	74,5	77,9	84,4	20,1	24,7	28,5	28,6	58,4	92,9	94,1	92,8	93,1	91,3	91	16,2	21,8	26,9	32	91,15
	LB	39,4	30,6	21,2	17,7	12,4	6,6				16,1	17,7	0,1	0	0	0	0,1	0,1				0	0
	SNR							0	0	0	0,1	0							0	0	0	0,2	0
	Resid	5,6	7,6	5,3	7,9	9,6	9	79,9	75,3	71,5	55,2	9,3	7	5,9	7,2	6,9	8,6	9	83,8	78,2	73,1	67,8	8,8
LCModel	Subject	60	63,4	69,2	69,7	70,1	77,2	28,6	36,5	37,2	30,2	61,7	58,1	63,3	66,3	74,3	84,7	83,7	35,2	32,2	42,8	34,3	60,7
	LB	10,7	10,5	12	12,6	12,9	9,8				10,6	10,7	13	9,7	7,1	4,7	1,9	0,7				3,9	4,7
	SNR							2,8	0,1	1,9	0,4	1,15							2	0,6	0	0,5	0,55
	Resid	29,3	26,1	18,8	17,7	17	13	68,6	63,3	60,9	58,8	27,7	28,9	27	26,6	21,1	13,4	15,6	62,8	67,2	57,2	61,2	27,95
Osprey	Subject	45,4	54,5	60,9	67,8	73,9	79,4	22,9	24,9	48,9	31,7	51,7	43,8	52,9	59,6	72	75	83,2	26,5	24,6	35,4	30,6	48,35
	LB	4,9	4,1	3,5	2	1,6	0,9				2,2	2,2	9,9	8	6,3	3,8	3,1	1,1				3,9	3,9
	SNR							0,1	0	0	0,3	0,05							0	0	0,7	0	0
	Resid	49,7	41,4	35,6	30,2	24,5	19,7	77	75,1	51,1	65,8	45,55	46,3	39,2	34,1	24,2	21,9	15,7	73,5	75,4	63,9	65,5	42,75
spant	Subject	43,2	41,2	42,9	45,9	48,6	57,4	34,9	30,9	56,6	21,6	43,05	70,3	64,8	67,7	66,2	67,6	73,4	38,2	35,6	33,8	39,2	65,5
	LB	31,5	34	32,8	30,3	25,4	19,8				25,7	30,3	2,7	4,1	4,5	3,8	2,4	1,9				2,5	2,7
	SNR							1,9	2,9	0	1,6	1,75							2,3	2,7	0,2	1,9	2,1
	Resid	25,3	24,8	24,3	23,8	26	22,9	63,1	66,2	43,4	51,1	25,65	27	31,1	27,8	30	30,1	24,7	59,5	61,8	66	56,4	30,6
Tarquin	Subject	56,4	62,2	70,5	70,7	76,2	81,2	22,5	29,6	41,8	30,9	59,3	68	72,4	79,9	83,4	84,7	88	32,4	32,4	54,2	43,6	70,2
	LB	24,9	21,2	15	14,5	10,7	6,9				12,5	14,5	8,6	7	4,5	2,4	1,9	0,4				2,4	2,4
	SNR							2,1	4,7	2	3,1	2,6							1,2	2,3	0	1,6	1,4
	Resid	18,7	16,7	14,5	14,8	13	12	75,4	65,7	56,2	53,5	17,7	23,4	20,6	15,6	14,2	13,3	11,6	66,3	65,4	45,8	52,3	22

Supplementary Table 4 Variance Partition Coefficients (VPCs) for GABA+ estimated from filtered spectra at specified SNR/Line Broadening levels; all VPCs quoted as percentages.

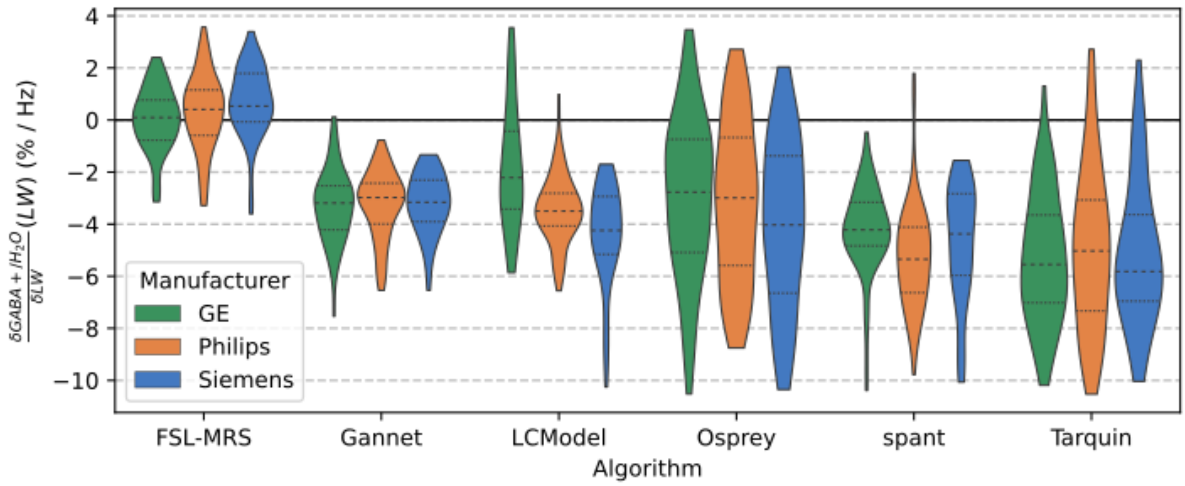

Supplementary Figure 6 LLWF (rate of change in GABA+ estimate in relation to Lorentzian linewidth) for each subject, grouped by algorithm and scanner manufacturer.

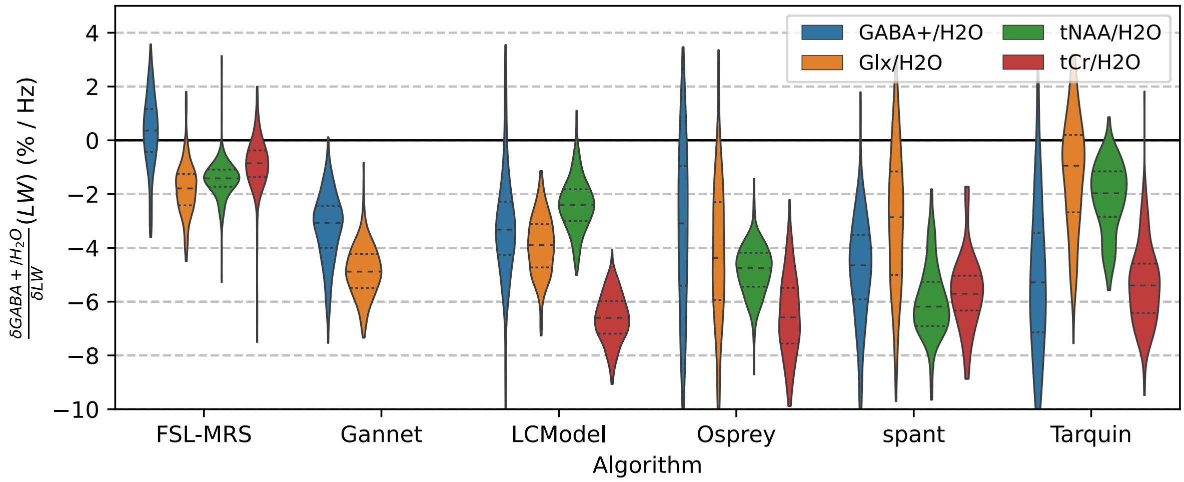

Supplementary Figure 7 LLWF (rate of change in concentration estimate in relation to Lorentzian linewidth) assessed for alternative metabolites

Algorithm 1	LLWF 1	Algorithm 2	LLWF 2	tstat	p	p_holm	sig
FSL-MRS	0.39 +- 1.4 %/Hz	Gannet	-3.25 +- 1.3 %/Hz	26,38	8,987E-91	1,258E-89	***
FSL-MRS	0.39 +- 1.4 %/Hz	LCModel	-3.16 +- 2.1 %/Hz	19,7	6,193E-60	7,432E-59	***
FSL-MRS	0.39 +- 1.4 %/Hz	Osprey	-3.05 +- 3.2 %/Hz	13,5	3,868E-32	4,254E-31	***
FSL-MRS	0.39 +- 1.4 %/Hz	Tarquin	-5.09 +- 2.9 %/Hz	24,16	1,927E-72	2,505E-71	***
FSL-MRS	0.39 +- 1.4 %/Hz	spant	-4.83 +- 2.0 %/Hz	30,29	2,505E-104	3,757E-103	***
Gannet	-3.09 +- 1.3 %/Hz	LCModel	-3.16 +- 2.1 %/Hz	-0,517	0,6055	1	
Gannet	-3.09 +- 1.3 %/Hz	Osprey	-3.05 +- 3.2 %/Hz	-0,834	0,4051	1	
Gannet	-3.09 +- 1.3 %/Hz	Tarquin	-5.09 +- 2.9 %/Hz	8,402	2,12E-15	1,696E-14	***
Gannet	-3.09 +- 1.3 %/Hz	spant	-4.83 +- 2.0 %/Hz	9,652	8,402E-20	8,402E-19	***
LCModel	-3.32 +- 2.1 %/Hz	Osprey	-3.05 +- 3.2 %/Hz	-0,4294	0,6679	1	
LCModel	-3.32 +- 2.1 %/Hz	Tarquin	-5.09 +- 2.9 %/Hz	7,823	5,296E-14	3,707E-13	***
LCModel	-3.32 +- 2.1 %/Hz	spant	-4.83 +- 2.0 %/Hz	8,386	7,514E-16	6,763E-15	***
Osprey	-2.99 +- 3.2 %/Hz	Tarquin	-5.09 +- 2.9 %/Hz	6,761	4,97E-11	2,982E-10	***
Osprey	-2.99 +- 3.2 %/Hz	spant	-4.83 +- 2.0 %/Hz	6,735	7,427E-11	3,714E-10	***
Tarquin	-5.26 +- 2.9 %/Hz	spant	-4.83 +- 2.0 %/Hz	-1,07	0,2856	1	

Supplementary Table 5 Groupwise statistics for GABA+/H_2O LLWF (see section 3.1.2)

Algorithm	Vendor 1	LLWF 1	Vendor 2	LLWF 2	tstat	p	p_holm	sig
FSL-MRS	G	0.09 +- 1.3 %/Hz	P	0.30 +- 1.5 %/Hz	-1,346	0,1804	1	
FSL-MRS	G	0.09 +- 1.3 %/Hz	S	0.75 +- 1.3 %/Hz	-3,028	0,003123	0,0468	*
FSL-MRS	P	0.52 +- 1.5 %/Hz	S	0.75 +- 1.3 %/Hz	-1,817	0,07202	0,8642	
Gannet	G	-3.19 +- 1.4 %/Hz	P	-3.25 +- 1.3 %/Hz	-0,03473	0,9724	1	
Gannet	G	-3.19 +- 1.4 %/Hz	S	-3.23 +- 1.2 %/Hz	-0,1225	0,9028	1	
Gannet	P	-2.98 +- 1.3 %/Hz	S	-3.23 +- 1.2 %/Hz	-0,1046	0,9168	1	
LCModel	G	-2.38 +- 2.4 %/Hz	P	-3.45 +- 1.3 %/Hz	4,85	5.323E-06	9E-05	***
LCModel	G	-2.38 +- 2.4 %/Hz	S	-4.27 +- 2.1 %/Hz	5,674	1,113E-07	2E-06	***
LCModel	P	-3.50 +- 1.3 %/Hz	S	-4.27 +- 2.1 %/Hz	2,491	0,01512	0,1965	
Osprey	G	-1.90 +- 3.0 %/Hz	P	-2.96 +- 3.2 %/Hz	1,066	0,2884	1	
Osprey	G	-1.90 +- 3.0 %/Hz	S	-3.99 +- 3.3 %/Hz	2,537	0,01281	0,1793	
Osprey	P	-3.02 +- 3.2 %/Hz	S	-3.99 +- 3.3 %/Hz	1,767	0,0807	0,8877	
Tarquin	G	-5.57 +- 2.4 %/Hz	P	-4.92 +- 3.1 %/Hz	-0,9852	0,3261	1	
Tarquin	G	-5.57 +- 2.4 %/Hz	S	-5.08 +- 2.8 %/Hz	-0,5701	0,5699	1	
Tarquin	P	-5.01 +- 3.1 %/Hz	S	-5.08 +- 2.8 %/Hz	0,3065	0,7598	1	
spant	G	-4.25 +- 1.7 %/Hz	P	-5.26 +- 2.0 %/Hz	3,24	0,001474	0,0236	*
spant	G	-4.25 +- 1.7 %/Hz	S	-4.67 +- 2.2 %/Hz	0,9545	0,3424	1	
spant	P	-5.35 +- 2.0 %/Hz	S	-4.67 +- 2.2 %/Hz	-1,576	0,1185	1	

Supplementary Table 6 Groupwise statistics for GABA+/H_2O LLWF, compared within algorithm across sites

D Supplementary Details: Synthetic Data

D.1 Synthetic data generation

Further to details in the main manuscript, section 2.3: Background signal derived by this approach is only meaningful on the original fit range (0.2-4.2 ppm); this has implications for the measurement of SNR, which often uses a region well beyond the normal fit range to assess the noise factor. Moreover, there is a risk of discontinuities (hence potential numeric instability) if the background function is not close to zero at the edge of the fit range. To avoid such discontinuities, a smooth spline was fit to the last 0.05 ppm at either end of the fit range, and extrapolated 0.10 ppm beyond the fit range. A pair of Hanning functions ($\cos^2 \frac{\pi(f-f_0)}{2a}$) were used to transition first from the signal to the smooth spline fit in the last 0.05 ppm, and then from the smooth spline fit to zero in the subsequent 0.10 ppm beyond the fit range.

D.2 Outcomes from fits to synthetic data

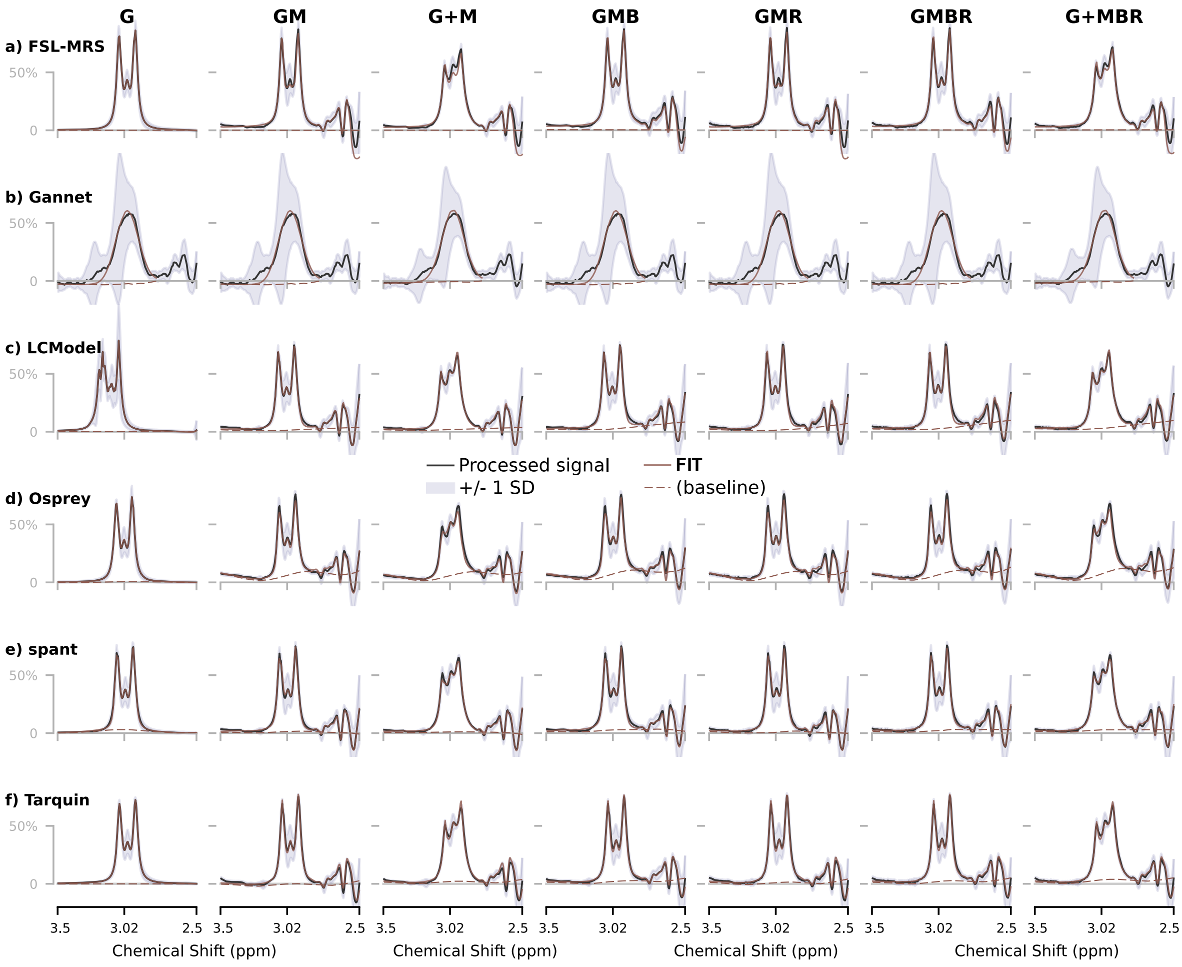

Supplementary Figure 8 Synthetic spectra and fits by various algorithms

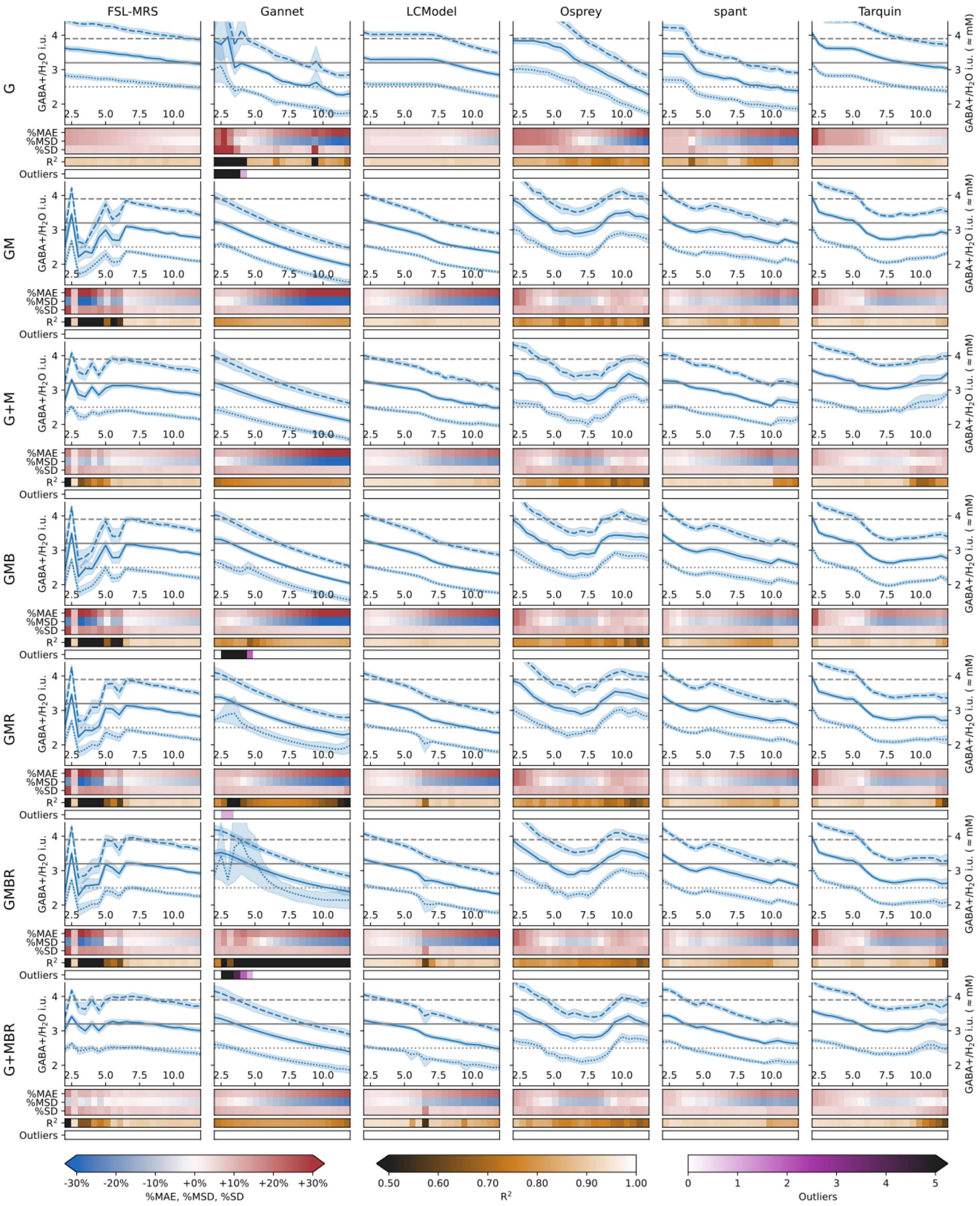

Supplementary Figure 9 GABA+/H2O estimates from each algorithm as a function of Lorentzian linewidth, based on synthetic data models of varying complexities, for nominal GABA+ concentrations as detailed in Section 2.3.

E Exploratory Analysis #1: spant baseline modelling

E.1 Background and Methods

The default fitting algorithm in spant is ABfit. A key component of this algorithm is the adaptive baseline model, in which the degree of baseline flexibility is automatically adjusted to match the complexity of the data[1]. As with other algorithms, this is inevitably a balancing act between a baseline that is too smooth, and one that is too flexible – with either scenario potentially leading to bias or increased variance in the fit outcome. Most pre-existing literature and documentation relating to spant at the time of this analysis focussed on non-edited spectra, such as may be obtained by short TE PRESS sequence. Early piloting suggested that default parameters suitable for such sequences may have allowed for too much baseline flexibility for GABA-edited datasets – as such, several different configurations of spant were applied to the in-vivo datasets to assess their relative suitability for GABA-edited spectra.

There are a number of options available for controlling ABfit's baseline modelling; two of these are `aic_smoothing_factor` and `bl_ed_ppm`:

- `aic_smoothing_factor` affects the criterion used for automatic estimation, with smaller values encouraging more flexible baselines, while remaining adaptive to any broad spectral features. Five different options for `aic_smoothing_factor` were assessed: 5 (default), 10, 20, 40 and 80, specifying increasingly firm baselines; these are subsequently denoted A05, A10, A20, A40 and A80 respectively.

- `bl_ed_pppm` specifies the baseline flexibility, in units of ED per ppm where ED is the "effective dimension" – analogous to the number of spline functions required per ppm; this is automatically determined except when `auto_bl_flex` (automatic baseline flexibility) is explicitly disabled. Three fixed values for `bl_ed_pppm` were tested (with `auto_bl_flex` disabled): 1.00, 1.67 and 2.00 ED/ppm, denoted F10, F17 and F20.

[1] These parameters are detailed at https://martin3141.github.io/spant/articles/abfit-baseline-opts.html

This analysis was performed using all in-vivo datasets from the main study, with processing and quality control as described in the main manuscript – including spectra with Lorentzian linebroadening, but not including those of experimentally degraded SNR.

To assess the relative suitability of each configuration, the determined baseline flexibility (`bl_ed_pppm`) and resultant signal-to-residual ratio (SRR) were inspected. Furthermore, GABA+ estimates were assessed for accuracy and consistency with other algorithms methods[2]. Exploiting the different expected GABA concentrations between grey and white matter[3–5], the degree of correlation between GABA estimates and voxel tissue fraction was used as an index of the accuracy of GABA estimation[6]. Robust (skipped)[7,8] Spearman correlation coefficients between voxel grey matter fraction and water-scaled GABA estimate without adjustment for tissue content (GABA+/$H2O_{noTC}$) were reported. Finally, intra-class correlation coefficients (ICCs) were calculated between each spant variant and the median outcome from all other algorithms, using a two-way mixed-effects model for single-rater consistency (ICC(3,1), implemented in pingouin[9]).

E.2 Results and Discussion

Outcomes for each of the tested configurations are presented in Supplementary Table 7. Using automatic baseline flexibility (A*nn*), the determined `bl_ed_pppm` factor was seen to clip at either edge of the allowable range (.502, 7.00) in a substantial proportion of cases; with A05 case (`aic_smoothing_factor`=5), the majority of estimates clipped to the predefined upper limit; in the A80 case, the majority clipped to the lower limit. The reported GABA.sd metric is suspiciously low in several cases (particularly with lower `bl_ed_pppm` values); as such, this metric is not considered further herein, but may warrant further investigation elsewhere. Signal-to-residual ratio (SRR) was generally higher in the cases with greater baseline flexibility, in particular A05. This is to be expected: more flexible baselines are more able to absorb signal fluctuations which would otherwise remain in the residuals. The intraclass correlation coefficient (ICC), indicating agreement with other algorithms, was highest in the fixed `bl_ed_pppm` cases (F*nn*), particularly F17 (`bl_ed_pppm`=1.67); this configuration is roughly equivalent to the 0.6 ppm baseline knot spacing adopted in both LCModel and Osprey, hence it is unsurprising that greatest agreement is seen in this case, where the baseline parameterisation is most similar. Using correlation between

GABA+/H2O$_{noTC}$ and tissue grey matter (GMcorr) as an indication of measurement accuracy, although subtle variation was seen, none of the configurations was found to be significantly more accurate than any other. The impact of line broadening, as assessed with the LLWF (see main manuscript section 2.5), was substantially reduced in magnitude as baseline flexibility was reduced.

Subset	parameter	A05	A10	A20	A40	A80	F10	F17	F20
Unfiltered only	GABA+/H2O	3.39 [3.02; 3.81]	3.42 [3.06; 3.84]	3.78 [3.33; 4.16]	3.87 [3.47; 4.42]	3.91 [3.48; 4.38]	3.86 [3.53; 4.37]	3.69 [3.42; 4.08]	3.56 [3.29; 3.95]
	aic_smoothing_factor	5	10	20	40	80	*N/A*	*N/A*	*N/A*
	bl_ed_pppm	7.00 [7.00; 7.00]	7.00 [4.09; 7.00]	1.35 [0.502; 5.89]	0.502 [0.502; 1.93]	0.502 [0.502; 0.503]	*1*	*1,67*	*2*
	SRR	26.8 [13.1; 148.]	17.1 [10.6; 23.4]	26.4 [9.98; 112.]	11.0 [8.15; 14.7]	10.5 [7.60; 14.0]	10.8 [7.52; 14.4]	11.7 [8.10; 15.5]	12.1 [8.45; 16.1]
	GABA.sd	1.35 [0.411; 4.45]	0.622 [0.350; 1.71]	0.0101 [6.62e-09; 1.27]	1.78e-08 [1.24e-8; 0.235]	1.75e-08 [1.2e-8; 2.7e-8]	2.33e-08 [1.39e-8; 0.001]	0.188 [0.0121; 0.266]	0.250 [0.178; 0.853]
	Gmcorr	0,364	0,396	0,346	0,377	0,4	0,363	0,368	0,372
	ICC	0.407 {0.290; 0.510}	0.438 {0.320; 0.540}	0.580 {0.480; 0.660}	0.641 {0.560; 0.710}	0.636 {0.550; 0.710}	0.711 {0.640; 0.770}	0.675 {0.600; 0.740}	0.665 {0.580; 0.730}
All (filtered and unfiltered)	GABA+/H2O	2.95 [2.25; 3.47]	2.98 [2.37; 3.50]	3.13 [2.57; 3.73]	3.49 [2.77; 4.11]	3.65 [2.99; 4.25]	3.64 [3.02; 4.32]	3.41 [2.95; 3.89]	3.26 [2.78; 3.74]
	bl_ed_pppm	7.00 [7.00; 7.00]	7.00 [5.89; 7.00]	4.92 [0.825; 7.00]	0.626 [0.502; 3.39]	0.502 [0.502; 1.61]	*1*	*1,67*	*2*
	SRR	44.7 [19.6; 198.]	24.3 [16.4; 35.4]	44.0 [15.0; 161.]	12.3 [9.14; 16.7]	10.2 [7.07; 13.8]	10.5 [6.23; 14.3]	12.1 [7.96; 15.9]	12.9 [8.84; 16.9]
	GABA.sd	0.740 [0.252; 2.55]	0.395 [0.215; 1.28]	0.478 [5.98e-05; 1.87]	2.38e-08 [8.62e-9; 0.381]	1.32e-08 [7.09e-9; 0.004]	1.72e-08 [7.81e-9; 0.0004]	0.108 [0.00415; 0.198]	0.167 [0.0878; 0.603]
	LLWF	-5.78 {-6.02; -5.52}	-5.69 {-5.95; -5.40}	-4.54 {-4.75; -4.32}	-3.00 {-3.27; -2.80}	-2.85 {-3.03; -2.53}	-3.10 {-3.29; -2.83}	-3.99 {-4.34; -3.75}	-4.35 {-4.54; -4.06}

Supplementary Table 7 Selected fitting performance metrics for spant, using a variety of baseline configurations. Values are reported as median with [10,90]-percentiles or {95% confidence interval}.

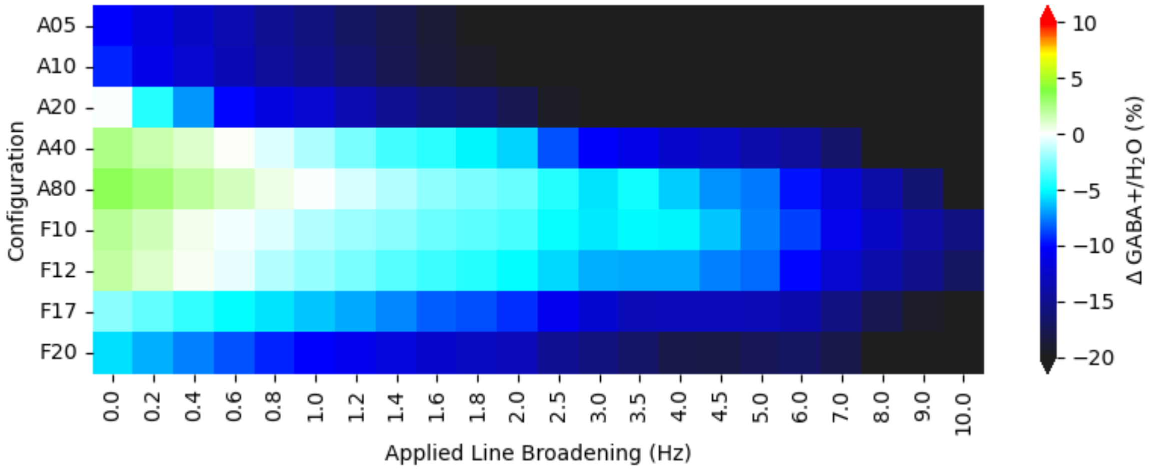

Supplementary Figure 10 Relative difference in GABA+ concentration reported by spant in different configurations, in relation to applied line broadening

Although certain configurations displayed better performance for individual metrics, or for individual sites' datasets, no single configuration was found to clearly outperform the others across a range of quality metrics in the general case. For settings where linewidth differences are not expected to be an issue, the default configuration (A05) remains a reasonable choice; in cases where linewidth changes may be of concern, a more firm baseline model should be adopted.

In the present study, configuration A20 was adopted (`aic_smoothing_factor`=20) for the main analysis: this configuration is seen to allow the Abfit algorithm to operate over its configured range (rather than clipping significantly at either limit), has a 'typical' baseline resolution similar to that of other methods, and gives moderately good outcomes across a range of quality metrics without optimising for any particular factor.

F Exploratory Analysis #2: FSL-MRS baseline modelling, optimization and lineshape

F.1 Background and Methods

FSL-MRS exhibited two noteworthy characteristics in the main analysis:

- For the filtered in-vivo datasets, fit outcomes showed notably lower linewidth dependency (for Lorentzian linebroadening) than for any of the other configurations assessed.
- Somewhat greater variance was seen for datasets of extremely low linewidth (particularly apparent in the simulated datasets)

To investigate the first and mitigate the second, several alternative configurations of FSL-MRS were assessed:

- Polynomial baseline order (`--baseline_order`) of -1 (no baseline), 1 (linear) and 2-7 (*n*th order polynomials) were assessed
- Different optimization algorithms (`--algo`) were evaluated: Truncated Newton (“Newton”) and full Metropolis Hastings (“MH”). In typical usage, the latter is initialised internally by the substantially faster Newton algorithm.
- Modelling without and with a separate metabolite group for NAA and NAAG (`--metab_groups NAA+NAAG`), to allow shift and broadening of those metabolites to be optimised separately from all other components (hence allowing a slight frequency shift to mitigate the observed instability at very low linewidths)
- Comparing the `--lorentzian` lineshape model against the default Voigt lineshape model: the former may be a closer match to the line broadening applied in the present analysis, whereas the latter is expected to more fully reflect the different broadening factors present in in-vivo data.

As with the spant baseline exploration, the same in-vivo datasets, processing and quality control as described in the main manuscript were used – including spectra with Lorentzian linebroadening spectra, but not including those of experimentally degraded SNR.

F.2 Results and Discussion

Lower-order baseline models (below 5th order) exhibit the lowest linewidth dependence for moderate line-broadening factors, up to about 5 Hz; slightly increased linewidth dependence was seen with higher-order baseline models. With higher order baselines ascribing progressively more signal to baseline, the default 2nd order baseline remains a reasonable choice for scenarios where moderate linewidth differences may be encountered. Adopting a pure Lorentzian lineshape model resulted in only marginal differences, with a tendency towards fractionally higher estimates and perhaps a slightly reduced linewidth dependence for moderate linewidths, in cases where a baseline was modelled (not statistically significant).

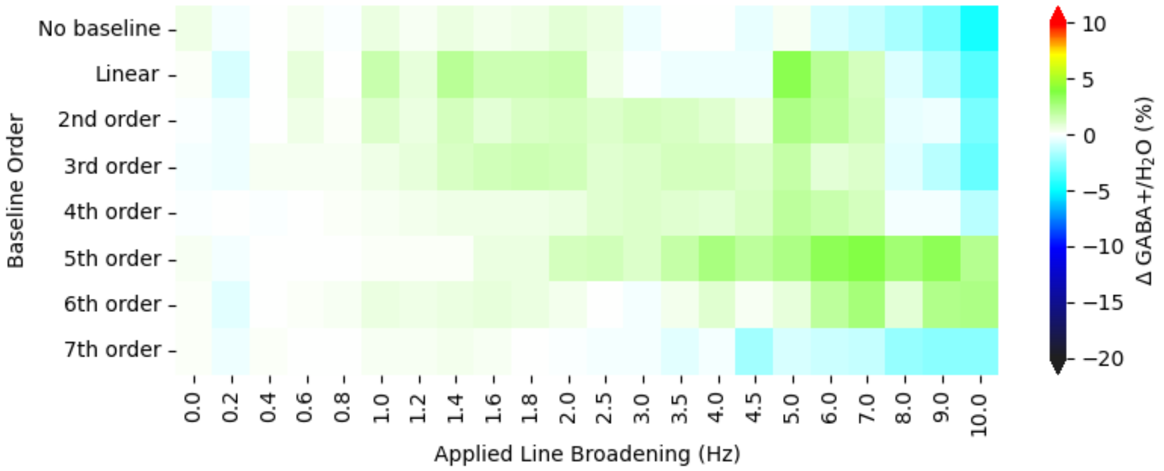

Supplementary Figure 11 Relative difference in GABA+ concentration reported by FSL-MRS with differing baseline configurations and default Lorentzian + Gaussian linewidth model, in relation to applied line broadening

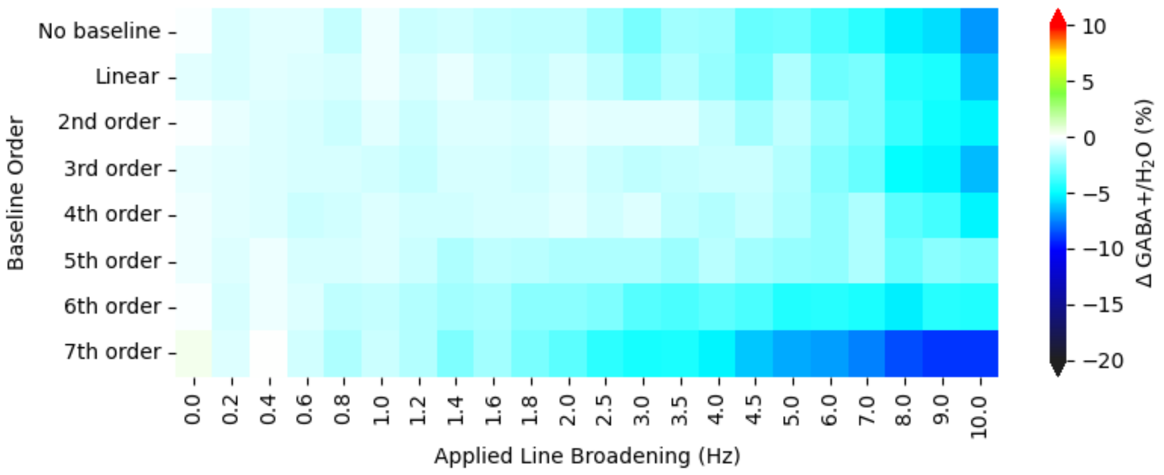

Supplementary Figure 12 Relative difference in GABA+ concentration reported by FSL-MRS with differing baseline configurations and pure Lorentzian lineshape model, in relation to applied line broadening

The GABA+ model sub-parts (GABA and MM3co) exhibit complementary behaviours which combine to give a relatively flat GABA+ response; estimates for these constituent parts are more stable (in relation to linewidth) when a 2nd order baseline is modelled. However, at lower linewidths (<2 Hz broadening) some additional variance in the estimates appears, particularly in cases where a baseline is modelled. This is reduced (but not entirely eliminated) when using the full MH model, which additionally exhibits slightly reduced linewidth dependence.

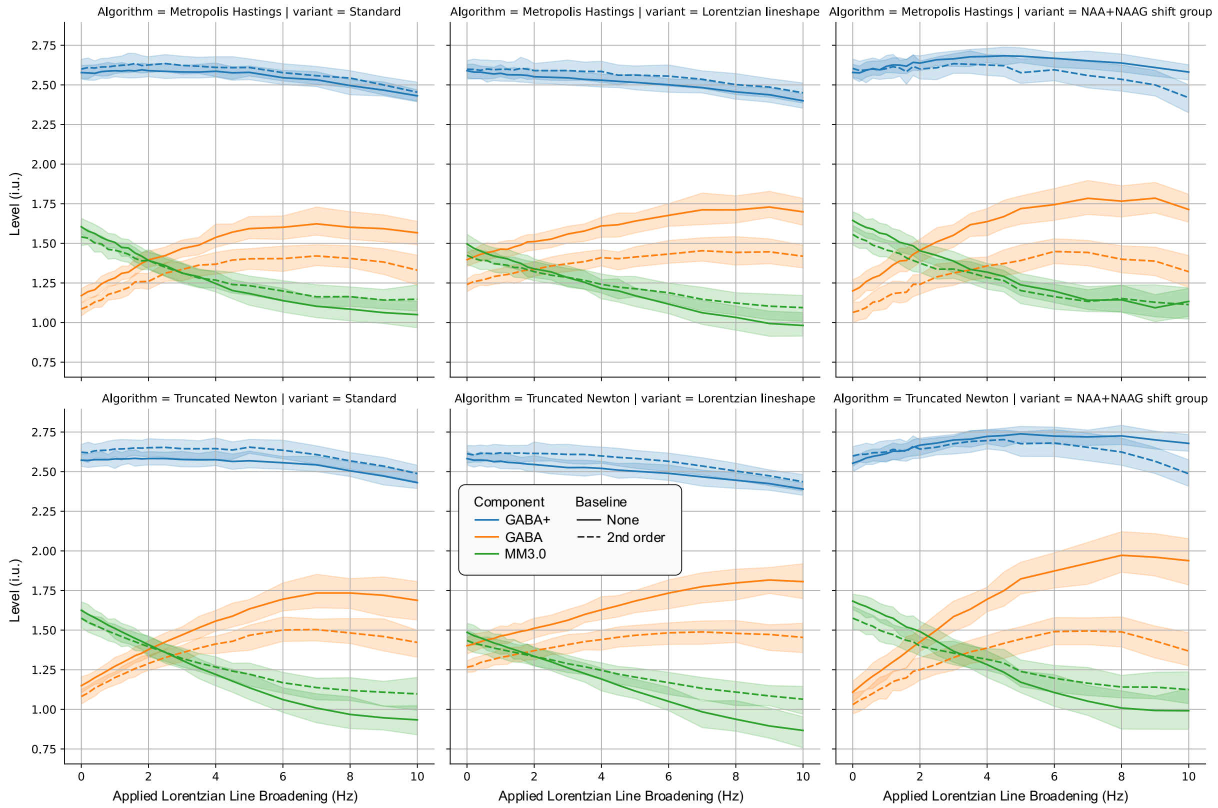

Supplementary Figure 13 Relative GABA+ and partial GABA/MM3co estimates from FSL-MRS under varying linewidth models (Voigt or Lorentzian), differing baseline configurations (2nd order or None), and differing optimization strategies (truncated Newton or full Metropolis-Hastings)

Baseline Model	algo	Variant	Unfiltered only GABA+/H2O	GMcorr	ICC	All (filtered and unfiltered) GABA+/H2O	LLWF
No baseline	Newton		2.60 [2.16; 2.92]	0,356	0.754 {0.690; 0.810}	2.58 [2.12; 2.97]	0.188 {0.0755; 0.349}
Linear	Newton		2.59 [2.14; 3.03]	0,306	0.854 {0.810; 0.890}	2.60 [2.11; 3.08]	0.396 {0.309; 0.630}
2nd order	Newton		2.58 [2.14; 3.14]	0,251	0.860 {0.820; 0.890}	2.61 [2.10; 3.18]	0.407 {0.184; 0.602}
3rd order	Newton		2.57 [2.10; 3.16]	0,296	0.844 {0.800; 0.880}	2.60 [2.04; 3.20]	0.217 {0.0221; 0.472}
4th order	Newton		2.58 [2.07; 3.12]	0,327	0.833 {0.790; 0.870}	2.59 [2.04; 3.19]	0.376 {0.110; 0.470}
5th order	Newton		2.59 [2.12; 3.12]	0,313	0.775 {0.720; 0.820}	2.61 [2.09; 3.19]	0.309 {0.0703; 0.482}
6th order	Newton		2.58 [2.06; 3.09]	0,389	0.704 {0.630; 0.770}	2.60 [2.06; 3.15]	0.189 {-0.0378; 0.425}
7th order	Newton		2.59 [2.05; 3.11]	0,413	0.695 {0.620; 0.760}	2.57 [2.03; 3.12]	**-0.0392 {-0.245; 0.0781}**
No baseline	Newton	NAA+NAAG	2.56 [2.15; 3.00]	0,432	0.719 {0.650; 0.780}	2.71 [2.22; 3.28]	2.42 {2.05; 2.76}
Linear	Newton	NAA+NAAG	2.53 [2.10; 3.10]	0,294	0.807 {0.750; 0.850}	2.71 [2.12; 3.44]	2.35 {1.91; 2.86}
2nd order	Newton	NAA+NAAG	2.52 [2.09; 3.27]	0,271	0.843 {0.800; 0.880}	2.65 [2.05; 3.47]	2.04 {1.70; 2.56}
3rd order	Newton	NAA+NAAG	2.54 [2.05; 3.32]	**0,237**	0.860 {0.820; 0.890}	2.66 [2.07; 3.49]	2.57 {2.07; 2.84}
4th order	Newton	NAA+NAAG	2.56 [2.04; 3.26]	0,281	0.829 {0.780; 0.870}	2.67 [2.11; 3.50]	2.90 {2.37; 3.29}
5th order	Newton	NAA+NAAG	2.55 [2.03; 3.18]	0,278	0.812 {0.760; 0.850}	2.67 [2.11; 3.49]	**2.94 {2.23; 3.37}**
6th order	Newton	NAA+NAAG	2.55 [2.02; 3.21]	0,364	0.735 {0.670; 0.790}	2.71 [2.10; 3.48]	2.46 {2.01; 3.00}
7th order	Newton	NAA+NAAG	2.55 [2.06; 3.25]	0,376	**0.665 {0.580; 0.730}**	2.71 [2.08; 3.47]	1.92 {1.54; 2.45}
No baseline	Newton	Lorentzian	2.60 [2.17; 2.96]	0,309	0.715 {0.640; 0.770}	2.55 [2.10; 2.93]	-0.728 {-0.874; -0.574}
Linear	Newton	Lorentzian	2.59 [2.14; 3.02]	0,304	0.849 {0.810; 0.880}	2.57 [2.08; 3.02]	-0.250 {-0.419; -0.159}
2nd order	Newton	Lorentzian	2.60 [2.14; 3.11]	0,273	**0.869 {0.830; 0.900}**	2.57 [2.07; 3.12]	-0.174 {-0.306; 0.103}
3rd order	Newton	Lorentzian	2.59 [2.09; 3.18]	0,266	0.858 {0.820; 0.890}	2.56 [2.01; 3.18]	-0.169 {-0.344; 0.0912}
4th order	Newton	Lorentzian	2.59 [2.08; 3.14]	0,336	0.839 {0.790; 0.870}	2.57 [2.01; 3.17]	-0.259 {-0.487; -0.120}
5th order	Newton	Lorentzian	2.59 [2.12; 3.06]	0,325	0.799 {0.750; 0.840}	2.56 [2.02; 3.11]	-0.597 {-0.826; -0.420}
6th order	Newton	Lorentzian	2.60 [2.13; 3.05]	0,423	0.729 {0.660; 0.790}	2.54 [2.01; 3.04]	-1.08 {-1.27; -0.862}
7th order	Newton	Lorentzian	2.61 [2.11; 3.07]	0,435	0.732 {0.660; 0.790}	2.50 [1.95; 2.99]	-1.59 {-1.80; -1.39}
No baseline	MH		2.60 [2.15; 2.96]	0,323	0.807 {0.750; 0.850}	2.58 [2.12; 2.98]	0.220 {0.00176; 0.399}
2nd order	MH		2.56 [2.09; 3.15]	0,259	0.834 {0.790; 0.870}	2.58 [2.03; 3.18]	0.276 {0.118; 0.527}
No baseline	MH	NAA+NAAG	2.55 [2.18; 3.04]	0,414	0.764 {0.700; 0.810}	2.67 [2.18; 3.25]	1.58 {1.27; 1.95}
2nd order	MH	NAA+NAAG	2.57 [2.01; 3.22]	0,245	0.833 {0.790; 0.870}	2.58 [1.98; 3.44]	1.23 {0.741; 1.78}
No baseline	MH	Lorentzian	2.60 [2.20; 3.01]	0,368	0.719 {0.650; 0.780}	2.55 [2.10; 2.95]	-0.526 {-0.679; -0.391}
2nd order	MH	Lorentzian	2.58 [2.06; 3.15]	0,254	0.831 {0.780; 0.870}	2.56 [2.01; 3.15]	-0.0440 {-0.356; 0.168}

Supplementary Table 8 Quality metrics for FSL-MRS modelling outcomes with differing baseline and algorithm options

G Exploratory Analysis #3: LCModel baseline knot spacing

G.1 Background and Methods

To assess the impact of baseline flexibility on linewidth dependence in LCModel, spectra were fit using a range of different baseline configurations: no baseline (`NOBASE=T`), and spline knot spacings from 0.1 to 4.0 ppm (in steps of 0.1 ppm between 0.1 to 1.2 ppm, steps of 0.2 ppm between 1.2 and 2.0 ppm, and in steps of 0.5 ppm from 2.0 up to 4.0 ppm). As previously, in-vivo data sets were fit after processing as described in the main manuscript, including spectra with Lorentzian linebroadening but not including those of experimentally degraded SNR.

G.2 Results and Discussion

GABA+ estimates obtained when no baseline was modelled were significantly elevated relative to those obtained when also using a baseline model. Unsurprisingly, excessively flexible baseline models (< 0.4 ppm knot spacing) resulted in significantly stronger linewidth-dependent biases. Firmer baseline models (>0.4 ppm) led to lower linewidth-dependent bias, particularly in the 0.5 – 1.0 ppm knot spacing range; this is compatible with previous recommendations for knot spacing[2,10] in the order of 0.55-0.6 ppm.

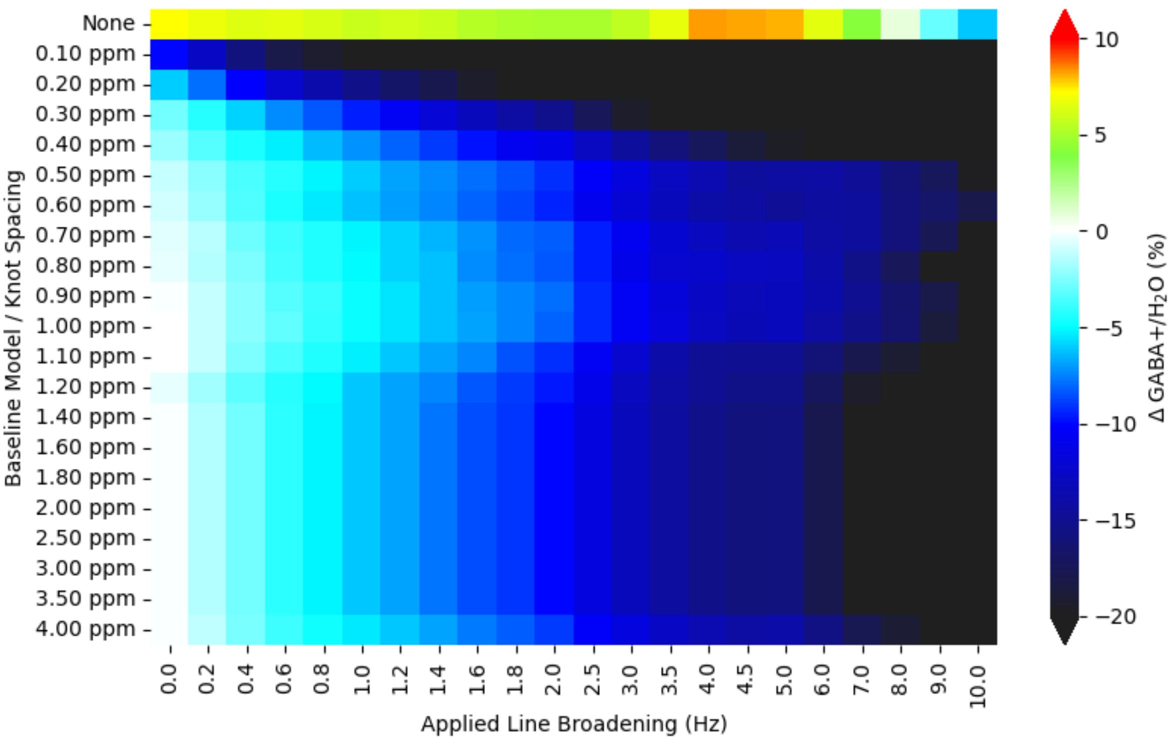

Supplementary Figure 14 Relative difference in GABA+ concentration reported by LCModel with different baseline knot spacings, in relation to applied line broadening

Baseline Knot Spacing	GABA+/H2O	Unfiltered only<br>GABA+ %SD	GMcorr	ICC	All (filtered and unfiltered)<br>GABA+/H2O	GABA+ %SD	LLWF
None	3.52 [3.16; 3.89]	10.5 [7.52; 16.9]	**0,305**	0.630 {0.540; 0.700}	3.45 [2.78; 4.17]	10.5 [4.00; 21.1]	**-1.16 {-1.47; -0.787}**
0.10 ppm	2.68 [2.36; 3.05]	13.6 [10.3; 20.8]	0,352	**0.432 {0.320; 0.530}**	2.34 [1.33; 3.22]	**12.6 [6.01; 25.6]**	-6.75 {-7.28; -6.05}
0.20 ppm	2.87 [2.57; 3.18]	11.7 [8.64; 17.5]	0,321	0.578 {0.480; 0.660}	2.52 [1.60; 3.34]	10.4 [5.78; 21.4]	**-7.61 {-8.14; -7.03}**
0.30 ppm	3.10 [2.83; 3.42]	10.9 [7.88; 16.4]	0,332	0.675 {0.600; 0.740}	2.71 [1.76; 3.44]	9.95 [5.00; 20.6]	-6.71 {-7.38; -6.26}
0.40 ppm	3.15 [2.89; 3.48]	10.7 [7.30; 16.1]	0,334	0.705 {0.630; 0.770}	2.89 [2.23; 3.53]	9.67 [4.84; 20.6]	-4.62 {-5.06; -4.26}
0.50 ppm	3.19 [2.92; 3.53]	10.5 [7.00; 16.2]	0,32	0.744 {0.680; 0.800}	2.97 [2.36; 3.59]	**9.64 [4.59; 21.0]**	-3.76 {-3.96; -3.52}
0.60 ppm	3.19 [2.92; 3.53]	10.6 [7.10; 16.0]	0,35	0.745 {0.680; 0.800}	2.97 [2.36; 3.60]	9.89 [4.78; 21.0]	-4.02 {-4.31; -3.70}
0.70 ppm	3.20 [2.94; 3.55]	10.5 [7.02; 15.6]	0,33	0.731 {0.660; 0.790}	2.99 [2.37; 3.62]	9.77 [4.82; 20.9]	**-3.63 {-4.10; -3.24}**
0.80 ppm	3.22 [2.92; 3.56]	10.5 [7.03; 15.8]	0,342	0.745 {0.680; 0.800}	2.98 [2.34; 3.61]	9.76 [4.65; 20.9]	-3.90 {-4.18; -3.52}
0.90 ppm	3.22 [2.92; 3.55]	10.4 [7.09; 15.7]	0,352	**0.747 {0.680; 0.800}**	3.00 [2.37; 3.62]	9.69 [4.50; 21.0]	-3.89 {-4.13; -3.40}
1.00 ppm	3.22 [2.91; 3.55]	10.4 [7.10; 16.0]	0,328	0.745 {0.680; 0.800}	2.99 [2.36; 3.62]	9.67 [4.32; 20.8]	-3.90 {-4.09; -3.47}
1.10 ppm	3.24 [2.84; 3.59]	10.4 [7.09; 16.5]	0,343	0.743 {0.680; 0.800}	2.96 [2.20; 3.67]	9.68 [4.09; 20.8]	-4.39 {-4.85; -3.97}
1.20 ppm	3.22 [2.85; 3.52]	10.6 [7.04; 16.7]	0,342	0.741 {0.670; 0.800}	2.93 [2.21; 3.62]	9.95 [4.26; 21.0]	-4.57 {-4.79; -4.08}
1.40 ppm	3.21 [2.86; 3.55]	10.5 [7.09; 16.1]	**0,364**	0.743 {0.680; 0.800}	2.93 [2.16; 3.64]	9.90 [4.00; 20.8]	-4.67 {-5.21; -3.97}
1.60 ppm	3.21 [2.86; 3.55]	10.5 [7.09; 16.1]	**0,364**	0.743 {0.680; 0.800}	2.93 [2.16; 3.64]	9.90 [4.00; 20.8]	-4.67 {-5.19; -3.98}
1.80 ppm	3.21 [2.86; 3.55]	10.5 [7.09; 16.1]	**0,364**	0.743 {0.680; 0.800}	2.93 [2.16; 3.64]	9.90 [4.00; 20.8]	-4.67 {-5.21; -3.98}
2.00 ppm	3.21 [2.86; 3.55]	10.5 [7.09; 16.1]	**0,364**	0.743 {0.680; 0.800}	2.93 [2.16; 3.64]	9.90 [4.00; 20.8]	-4.67 {-5.20; -4.11}
2.50 ppm	3.21 [2.86; 3.55]	10.5 [7.09; 16.1]	**0,364**	0.743 {0.680; 0.800}	2.93 [2.16; 3.64]	9.90 [4.00; 20.8]	-4.67 {-5.19; -3.98}
3.00 ppm	3.21 [2.86; 3.55]	10.5 [7.09; 16.1]	**0,364**	0.743 {0.680; 0.800}	2.93 [2.16; 3.64]	9.90 [4.00; 20.8]	-4.67 {-5.19; -3.98}
3.50 ppm	3.21 [2.86; 3.55]	10.5 [7.09; 16.1]	**0,364**	0.743 {0.680; 0.800}	2.93 [2.16; 3.64]	9.90 [4.00; 20.8]	-4.67 {-5.19; -3.98}
4.00 ppm	3.21 [2.93; 3.53]	10.5 [7.09; 15.8]	0,348	0.730 {0.660; 0.790}	2.96 [2.33; 3.59]	9.85 [4.00; 20.4]	-4.12 {-4.44; -3.80}

Supplementary Table 9 Quality metrics for LCModel modelling outcomes with differing baseline configurations

H Supplementary Methods

Further details on specific parameters adopted for each of the modelling algorithms are presented below; details in this section have been published previously[2], and are reproduced here for completeness. This supplements detail provided in the main article, Section 2.4. Section H.1 focusses on modelling of the GABA-edited difference spectrum, with adaptions for the edit-OFF case mentioned briefly in Section H.2 where applicable.

H.1 Difference spectrum modelling

H.1.1 FSL-MRS

FSL-MRS [11] models data as a linear combination of basis components in the frequency domain, using Bayesian statistics for optimization. Version 1.1.1 was used, with the standard basis set as described in section 2.2 and the recommended Metropolis-Hastings algorithm on the default fit range (0.2-4.2 ppm).

Default baseline model is a second-order polynomial fit over the defined fit range. To allow FSL-MRS to yield water-scaled estimates, NAA+NAAG was defined as an internal reference for basis set scaling, and fixed tissue content (50% white/50% grey matter, 0% CSF) were supplied; this scaling was subsequently reversed, before re-scaling with per-subject tissue content. Alternative baseline configurations and variations to the “shift group” and algo parameters are explored in Supplementary Section F.

Typical invocation of FSL-MRS for GABA-edited difference spectra was with the following parameters:

```
--basis ge_diff_8192_4000.basis
--data S12_GABA_68.diff.nii.gz
--h2o S12_GABA_68.ref.nii.gz
--output S12_GABA_68.diff
--overwrite
--algo MH
--TE 68
--TR 2.0
--tissue_frac .5 .5 0
--internal_ref NAA NAAG
--verbose
--report
--combine NAA NAAG
--combine Glu Gln GSH
--combine GABA MM3co
```

```
Defaults:
  --ppmlim:          (0.2, 4.2)
  --baseline_order:  2
  --metab_groups:    0
  --h2o_scale:       1.0
```

H.1.2 Gannet

Gannet [12] models data by fitting peaks in the frequency domain. Version 3.1 was used, with the 'GABA+Glx' model applied to the difference spectrum between 2.79 and 4.1 ppm. This model fits the GABA+ peak with a single Gaussian peak around 3.02 ± 0.05 ppm and Glx as a pair of Gaussian peaks at 3.71 ± 0.02 ppm and 3.79 ± 0.02 ppm, and includes terms to characterize the baseline. The Gannet baseline is modelled as a linear slope with sinusoid and cosine terms, periodic at 2.62 ppm (i.e., $A * (f - f_0) + B * \sin(\pi * f / 1.31 / 4) + C * \cos(\pi * f / 1.31 / 4)$ where f is the frequency (in ppm) and f_0 the offset to the first modelled Glx peak (±3.71 ppm).

The default Gannet processing pipeline applies zero-filling to 0.062 Hz spectral resolution, and line-broadening of 3 Hz. Both these steps were omitted in the present study.

H.1.3 LCModel

LCModel [13] was invoked with the following parameters for modelling GABA-edited difference spectra, to achieve a relatively stiff but non-zero cubic spline baseline model:

- `SPTYPE='mega-press-3'` sets the "special type" appropriately for MEGA-PRESS. This implies a flat baseline (`NOBASE=T`), the use of NAA @ 2.01 ppm as a reference for basis set scaling (`wsmet='NAA', wsppm=2.01`), and default correction factor for attenuation of NMR-visible water, ATTH2O = 0.43 (suitable for TE = 68 ms).

- `NOBASE=F` to re-enable baseline modelling (which is disabled by default for MEGA-PRESS when using `SPTYPE=mega-press-3`),

- `DKNTMN=0.6` to set the baseline knot spacing to 0.6 ppm (the default value is 0.15). This value was varied in the exploratory analysis, Supplementary Section G.
- `PPMST=4.2, PPMEND=0.2` model on the range 0.2-0.4 ppm…
- `PPMGAP(1,1)=1.95, PPMGAP(2,1)=1.2` …but exclude the 1.2-1.95 ppm range from modelling
- `DOWS=T` do water scaling
- `DOECC=F` disable ECC

For the simulated datasets containing only GABA signal, it was additionally necessary to disable frequency referencing: `DOREFS=F,F` and `PPMSHF=0.0`.

H.1.4 Osprey

Osprey [14] implements a frequency domain basis set fit; version 1.0.1.1 was used, with default fit and reconstruction settings: separate fit on the range 0.2-4.2 ppm, incorporating macromolecule and lipid components in the basis set but without adding a co-edited MM3 peak. Knot spacing for the spline baseline was set at 0.6 ppm. Although Osprey implements a number of soft constraint models, in this instance the MM3co basis set component was incorporated without additional constraints on amplitude.

H.1.5 spant

Spant was configured with automatic baseline flexibility `aic_smoothing_factor`=20, as detailed in Supplementary Section E.

H.1.6 Tarquin

A local build of Tarquin [15,16] v4.3.11 was used; processed data and per-vendor basis sets were supplied to Tarquin in the corresponding LCModel file formats.

NAA was used as a reference for basis set scaling. Appropriate TE, sweep width and centre frequency (`echo`, `fs`, `ft`) were specified on the command line. Automatic phasing and referencing (`auto_phase` and `auto_ref`) were disabled. The start point for analysis of the difference spectra (`start_pnt`) was set at 5 ms (calculated as 0.005 times the sample frequency). Eddy-current correction was disabled (`--water_eddy false`). HSVD was performed (per defaults) to remove residual water.

Typical parameters for Tarquin, modelling GABA-edited difference spectra:

```
tarquin
--format lcm
--input S12_GABA_68.diff.RAW
--input_w S12_GABA_68.diff.REF

--echo 0.068
--fs 5000
--ft 127714400
--start_pnt 25
--pul_seq mega_press

--auto_phase false
--auto_ref false
--water_eddy false

--w_att 0.76
--w_conc 35880

--ext_pdf true
--output_txt S12_GABA_68.diff.tarquin.txt
--output_csv S12_GABA_68.diff.tarquin.csv
--output_pdf S12_GABA_68.diff.pdf
--output_fit S12_GABA_68.tarquin.fit
--basis_lcm ge_diff_4096_5000.basis
```

H.2 Creatine Reference: Edit-OFF sub-spectrum modelling

For modelling of the edit-OFF sub-spectra (for all algorithms except for Gannet), a standard simulated basis set specific to each hardware vendor was adopted. These were derived for edit-OFF sub-spectra using a similar method to that used for the difference spectra (as detailed in Section H.1), incorporating a default set of basis components as defined within Osprey: ascorbate (Asc), aspartate (Asp), creatine (Cr), a negative correction term for the Creatine CH_2 singlet around 3.94 ppm (CrCH2), γ-aminobutyric acid (GABA), glycerophosphocholine (GPC), glutathione (GSH), Glutamine (Gln), Glutamate (Glu), water (H_2O), myo-inositol (Ins), lactate (Lac), N-acetyl aspartate (NAA), n-acetylaspartylglutamate (NAAG), phosphorylcholine (PCh), phosphocreatine (PCr), phosphoethanolamine (PE), scyllo-inositol (Scyllo), taurine (Tau), tyrosine (Tyros), and a series of macromolecule and lipid components: $MM_{0.9}$ $MM_{1.2}$ $MM_{1.4}$ $MM_{1.7}$ $MM_{2.0}$ $Lip_{0.9}$ $Lip_{1.3}$ $Lip_{2.0.}$

H.2.1 FSL-MRS

Edit-OFF sub-spectra were quantified with the standard edit-OFF basis set, using Cr+PCr as an internal reference for basis set scaling. For expedience, the default Newton optimisation algorithm was used; otherwise, all parameters were the same as for the difference case.

```
--basis             ge_off_8192_4000.basis
--data              S12_GABA_68.off.nii.gz
--h2o               S12_GABA_68.ref.nii.gz
--output            S12_GABA_68.off
--overwrite
--TE                68
--TR                2.0
--tissue_frac       .5 .5 0
--verbose
--report
--combine           NAA NAAG
--combine           Glu Gln GSH
--combine           Cr PCr
--combine           GPC PCh

Defaults:
--internal_ref      Cr PCr
--algo Newton
--ppmlim:           (0.2, 4.2)
--baseline_order:   2
--metab_groups:     0
--h2o_scale:        1.0
```

H.2.2 Gannet

In addition to the GABA+Glx model, Gannet independently fits a Lorentzian model to NAA around 2.01 ± 0.04 ppm, and a dual-Lorentzian model for Choline and Creatine, the latter centred at 3.02 ppm with a fixed 0.18 ppm separation, all from the edit-OFF sub-spectrum. A minor local modification was made to additionally yield water-referenced estimates from the existing Cr and NAA fits (usually only reported for those metabolites contained in the difference spectrum).

H.2.3 LCModel

Basic configuration was similar to that used for the difference spectra, with two key exceptions. `PPMGAP` was not defined – which implies fitting over the full defined fit range (0.2 - 4.2 ppm), the default mode of operation. `SPTYPE` and `DKNTMN` were also not defined, again implying a default fit: a regular spline baseline was modelled (with default knot spacing 0.15 ppm), Creatine was used for internal basis set scaling, and LCModel's default set of soft constraints were adopted.

H.2.4 Osprey

Edit-OFF sub-spectra were modelled with default parameters, in the same invocation of OspreyFit as for the difference spectra.

H.2.5 spant

Edit-OFF spectra were quantified using default parameters for spant.

H.2.6 Tarquin

Edit-OFF sub-spectra were quantified with HSVD water removal disabled (`--water_width 0`) but all other parameters equivalent to those used for the difference spectra.

```
tarquin

--format      lcm
--input       S12_GABA_68.off.RAW
--input_w     S12_GABA_68.off.REF

--echo        0.068
--fs          5000
--ft          127714400
--auto_phase  false
--auto_ref    false
--water_eddy  false
--water_width 0

--w_att       0.76
--w_conc      35880

--output_txt  S12_GABA_68.off.txt
--output_csv  S12_GABA_68.off.csv
--output_pdf  S12_GABA_68.off.pdf
--output_fit  S12_GABA_68.off.fit
--ext_pdf     true

--basis_lcm   ge_off_4096_5000.basis
```

I Supplementary References

1. Lin A, Andronesi O, Bogner W, et al. Minimum Reporting Standards for in vivo Magnetic Resonance Spectroscopy (MRSinMRS): Experts' consensus recommendations. *NMR Biomed*. Published online February 9, 2021. doi:10.1002/nbm.4484
2. Craven AR, Bhattacharyya PK, Clarke WT, et al. Comparison of seven modelling algorithms for γ-aminobutyric acid–edited proton magnetic resonance spectroscopy. *NMR Biomed*. Published online February 23, 2022. doi:10.1002/nbm.4702
3. Harris AD, Puts NAJ, Edden RAE. Tissue correction for GABA-edited MRS: Considerations of voxel composition, tissue segmentation, and tissue relaxations: Tissue Correction for GABA-Edited MRS. *J Magn Reson Imaging*. 2015;42(5):1431-1440. doi:10.1002/jmri.24903
4. Jensen JE, deB. Frederick B, Renshaw PF. Grey and white matter GABA level differences in the human brain using two-dimensional,J-resolved spectroscopic imaging. *NMR Biomed*. 2005;18(8):570-576. doi:10.1002/nbm.994
5. Mikkelsen M, Singh KD, Brealy JA, Linden DEJ, Evans CJ. Quantification of γ-aminobutyric acid (GABA) in ^{1} H MRS volumes composed heterogeneously of grey and white matter: GABA Quantification in Heterogeneous Volumes. *NMR Biomed*. 2016;29(11):1644-1655. doi:10.1002/nbm.3622
6. Rideaux R, Mikkelsen M, Edden RAE. Comparison of methods for spectral alignment and signal modelling of GABA-edited MR spectroscopy data. *NeuroImage*. 2021;232:117900. doi:10.1016/j.neuroimage.2021.117900
7. Pernet C, Wilcox R, Rousselet G. Robust Correlation Analyses: False Positive and Power Validation Using a New Open Source Matlab Toolbox. *Front Psychol*. 2013;3. doi:10.3389/fpsyg.2012.00606
8. Rousselet GA, Pernet CR. Improving standards in brain-behavior correlation analyses. *Front Hum Neurosci*. 2012;6. doi:10.3389/fnhum.2012.00119
9. Vallat R. Pingouin: statistics in Python. *J Open Source Softw*. 2018;3(31):1026. doi:10.21105/joss.01026
10. Zöllner HJ, Tapper S, Hui SCN, Barker PB, Edden RAE, Oeltzschner G. Comparison of linear combination modeling strategies for edited magnetic resonance spectroscopy at 3 T. *NMR Biomed*. 2022;35(1). doi:10.1002/nbm.4618
11. Clarke WT, Stagg CJ, Jbabdi S. FSL-MRS: An end-to-end spectroscopy analysis package. *Magn Reson Med*. 2021;85(6):2950-2964. doi:10.1002/mrm.28630
12. Edden RAE, Puts NAJ, Harris AD, Barker PB, Evans CJ. Gannet: A batch-processing tool for the quantitative analysis of gamma-aminobutyric acid-edited MR spectroscopy spectra: Gannet: GABA Analysis Toolkit. *J Magn Reson Imaging*. 2014;40(6):1445-1452. doi:10.1002/jmri.24478

13. Provencher SW. Estimation of metabolite concentrations from localizedin vivo proton NMR spectra. *Magn Reson Med*. 1993;30(6):672-679. doi:10.1002/mrm.1910300604

14. Oeltzschner G, Zöllner HJ, Hui SCN, et al. Osprey: Open-source processing, reconstruction & estimation of magnetic resonance spectroscopy data. *J Neurosci Methods*. 2020;343:108827. doi:10.1016/j.jneumeth.2020.108827

15. Reynolds G, Wilson M, Peet A, Arvanitis TN. An algorithm for the automated quantitation of metabolites in in vitro NMR signals. *Magn Reson Med*. 2006;56(6):1211-1219. doi:10.1002/mrm.21081

16. Wilson M, Reynolds G, Kauppinen RA, Arvanitis TN, Peet AC. A constrained least-squares approach to the automated quantitation of in vivo ^{1}H magnetic resonance spectroscopy data: Automated Quantitation of In Vivo ^{1}H MRS Data. *Magn Reson Med*. 2011;65(1):1-12. doi:10.1002/mrm.22579
